# The curcumin analogue PAC has potent anti-anaplastic thyroid cancer effects

**DOI:** 10.1038/s41598-023-30888-2

**Published:** 2023-03-14

**Authors:** Mai Al-Mohanna, Noura N. Alraouji, Samiah A. Alhabardi, Falah Al-Mohanna, Basem Al-Otaibi, Ibrahim Al-Jammaz, Abdelilah Aboussekhra

**Affiliations:** 1 Department of Molecular Oncology, King Faisal Specialist Hospital and Research Center, MBC # 03, PO Box 3354, Riyadh, 11211 Kingdom of Saudi Arabia; 2 Department of Pharmaceutics, College of Pharmacy, King Saud University, Riyadh, 11451 Kingdom of Saudi Arabia; 3 Department of Comparative Medicine, King Faisal Specialist Hospital and Research Center, Riyadh, 11211 Kingdom of Saudi Arabia; 4 Department of Cyclotron and Radiopharmaceuticals, King Faisal Specialist Hospital and Research Center, Riyadh, 11211 Kingdom of Saudi Arabia

**Keywords:** Biochemistry, Cancer, Drug discovery

## Abstract

Anaplastic thyroid carcinoma (ATC) is the rarest type of thyroid cancer, but is the common cause of death from these tumors. The aggressive behavior of ATC makes it resistant to the conventional therapeutic approaches. Thus, the present study was designed to evaluate the anti-ATC efficacy of the piperidone analogue of curcumin (PAC). We have shown that PAC induces apoptosis in thyroid cancer cells in a time-dependent fashion through the mitochondrial pathway. Immunoblotting analysis revealed that PAC suppressed the epithelial-to-mesenchymal transition (EMT) process in ATC cells by upregulating the epithelial marker E-cadherin and reducing the level of the mesenchymal markers N-cadherin, Snail, and Twist1. This anti-EMT effect was confirmed by showing PAC-dependent inhibition of the proliferation and migration abilities of ATC cells. Furthermore, PAC inhibited the AKT/mTOR pathway in ATC cells. Indeed, PAC downregulated mTOR and its downstream effectors p70S6K and 4E-BP1 more efficiently than the well-known mTOR inhibitor rapamycin. In addition to the promising in vitro anticancer efficacy, PAC significantly suppressed the growth of humanized thyroid tumor xenografts in mice. Together, these findings indicate that PAC could be considered as promising therapeutic agent for anaplastic thyroid carcinomas.

## Introduction

Thyroid cancers (TCs), the most common endocrine malignancies, are known to develop as either benign or malignant. Based on pathological characteristics thyroid cancer can be classified into three classes, papillary thyroid cancer (PTC), follicular thyroid cancer (FTC), and anaplastic thyroid cancer (ATC)^1^. Among these three TCs, the ATC is known as undifferentiated with high aggressiveness and invasiveness properties. Indeed, patients with ATC survive only four to six months after being diagnosed^2^.

While surgery remains the mainstay treatment approach for TCs, combination therapeutic approaches including hyper-fractionated accelerated external beam radiotherapy, followed by combination of chemotherapies with or without palliative care have been suggested as first-line treatments of ATC patients^3^. Due to the failure of single-drug chemotherapy, a combination of two or more agents such as cisplatin, docetaxel, doxorubicin, paclitaxel, and pegfilgrastim is administered to ATC patients. However, the second-line treatment comprises more targeted options such as anti-angiogenic drugs, tyrosine kinase inhibitors, and hyperactive BRAF, mTOR, and/or BCR-ABL targeted agonists and multi-kinase inhibitors^2,3^.

Several dietary components are of particular interest as anti-cancer molecules, and the most common examples are curcumin, epigallocatechin-3-gallate (EGCG), genistein, and resveratrol^4^. Allegri et al.^5^ evaluated the anticancer capacity of the aforementioned compounds against ATC and found curcumin to be the best anti-ATC agent. Curcumin, the main component of the spice turmeric (*Curcuma longa*), is a polyphenol molecule with numerous medicinal values. Curcumin is well known for its great and specific in vitro and in vivo anti-cancer aptitudes against cancer in breast, colon and rectum, head and neck, pancreas, prostate, and thyroid. However, despite the clear therapeutic benefits, curcumin has some limitations, such as low bioavailability and quick metabolism, which significantly reduced its clinical effectiveness^6^.

Aiming at overcoming the pharmacokinetics limitations, while keeping the native therapeutic effects and safety of curcumin, several curcumin analogues were synthecized. Interestingly, compared to the native one, few analogues showed better activity, while others exhibited similar or less performance^7^. The Piperidone Analogue of Curcumin (PAC)^8^ is a novel curcumin analogue synthesized by Youseff et al.^9^. Until now, the anticancer activity of PAC has been evaluated against breast cancer^10,11^, colon cancer^12^, and oral cancer^8^. Importantly, PAC exhibited better anti-cancer activity, greater biodistribution, and longer bioavailability compared to curcumin^10,11^. Thereby, we have studied in the present study the anti-cancer capacity of PAC against ATC both in vitro and in vivo.

## Materials and methods

### Cells and cell culture

CAL-62 cells were purchased from ATCC and were authenticated using short tandem repeat profiling by ATCC, propagated, expanded, and frozen immediately into numerous aliquots after arrival. The revived cells were utilized within 10 to 12 passages and not exceeding a period of 3 months, and were cultured following the instructions of the company. Cells were regularly screened for mycoplasma contamination using MycoAlert Mycoplasma Detection Kits (Lonza, Basel, Switzerland). All supplements were obtained from Gibco (Grand Island, NY). Cells were maintained at 37 °C in a humidified incubator with 5% CO2. PAC^8^ is a novel curcumin analogue developed by Youseff et al.^9^. The molecule is synthesized at King Faisal Specialist Hospital & Research Center (KFSH&RC) and storred at − 20 °C either as powder or diluted in DMSO at 10 mM.

### Cell migration and proliferation

Cell proliferation and migration were assessed using the real-time cell analyzer (RTCA) system fellowing the instructions of the manufacturer. For migration, 1 × 10^4^ cells in serum-free medium were added to the upper wells of the CIM plate, while the complete medium was added as a chemoattractant to the lower chamber wells. The migration ability was assessed during 24 h. For proliferation, cells (1 × 10^4^) were seeded in an E-plate with complete medium and the proliferation capacity was assessed for 72 h. Each assay was performed in triplicate.

### Apoptosis analysis by annexin V/flow cytometry

Cells were harvested, centrifuged and stained with propidium iodide (PI) and Alexa Flour 488 annexin V (Molecular Probes, Eugene), and then were analysed by flow cytometry.

### Cellular lysate preparation and immunoblotting

This has been performed as previously described^13^. Antibodies directed against N-cadherin, and Twist-1 were purchased from Abcam (Cambridge, MA); Cyclin D1 (HD11), β-actin (C-11), p70 S6K (H-9), p-p70 S6K (A-6) and 4E-BP1 A (P-1) were purchased from Santa Cruz (Santa Cruz, CA). Cleaved caspase-3 (Asp175), Cleaved caspase-9 (Asp 315), Cleaved-PARP-1 (ASP 214), Snail (C15D3), mTOR (7C10), P-mTOR (Ser2448, D9C2), GAPDH, E-cadherin (24E10), Akt and P-Akt (193H12) were purchased from Cell Signaling (Danvers, MA, USA).

After protein transfer, the memranes were cut to 2 or 3 pieces based on the molecular weight to allow simultaneous use of different antibodies corresponding to proteins with different molecular weights.

### Quantification of protein expression level

The protein signal intensity of each band was determined using ImageQuant TL software (GE Healthcare). Next, dividing the obtained value of each band by the value of the corresponding internal control allowed a correction of the loading differences. The fold change in the protein levels was determined by dividing the corrected values by that of the control.

### Tumor xenografts

Animal experiments were approved by the King Faisal Specialist Hospital and Research Center Institutional Animal Care and Use Committee (ACUC) under RAC# 2140031, and were conducted according to relevant national and international guidelines. Thyroid cancer xenografts were created in nude mice (n = 6) by subcutaneous injection of CAL62 cells (5 × 10^6^). When tumors reached a reasonable volume, the animals were randomized into two treatment groups to receive intraperitoneal (i.p.) injections of PAC (100 mg/kg) or the same volume of dimethyl sulfoxide (DMSO) every day for 25 days. Tumor volumes were measured regularly using a caliper and following the formula (Length × Width × Depth). The euthanasia was performed using a CO2 chamber followed by cervical dislocation.

### Statistical analysis

Statistical analysis was performed by student’s *t*-test and *P* values of 0.05 and less were considered as statistically significant.

### Statements

1. All experimental protocols were approved by the KFSH&RC Basic Research Committee under the RAC proposal # 2140031.
2. The study is reported in accordance with ARRIVE guidelines.

## Results

### PAC induces apoptosis in anaplastic thyroid cancer cells

**Figure 1:**
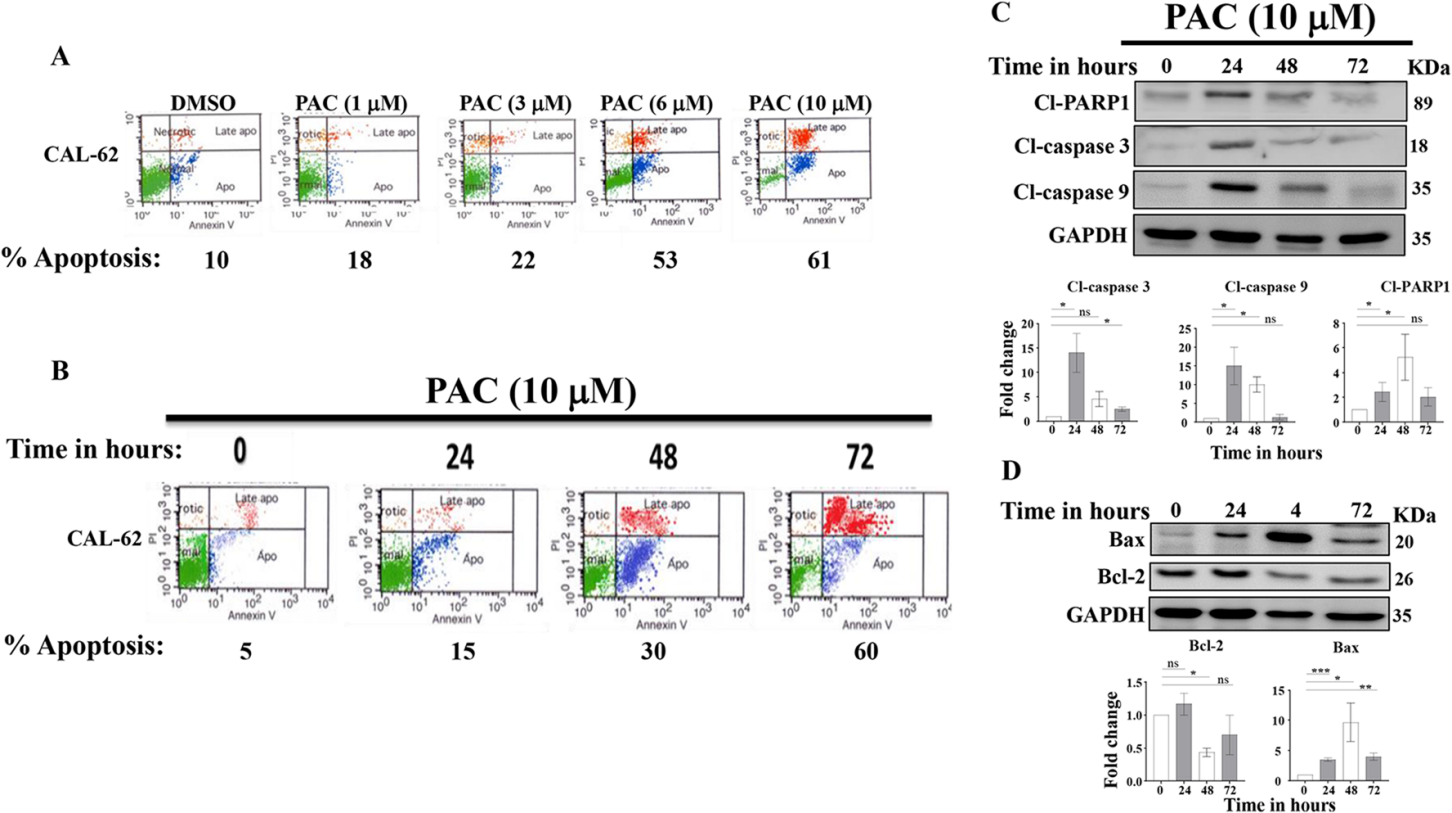
PAC induces apoptosis in anaplastic thyroid cancer cells. (**A**) Thyroid cancer cells were either sham-treated (DMSO ≤ 1%) or challenged with the indicated doses of PAC for 72 h, and then the proportion of apoptosis was analyzed by Annexin V/PI-flow cytometry. Flow charts. (**B**) CAL-62 cells were challenged with PAC for the indicated periods of time, and then the proportion of apoptotic cells was analyzed by Annexin V/PI-flowCytometry. (**C**, **D**) CAL-62 cells were treated with PAC (10 µM) for the indicated periods of time, and then cell lysates were prepared and used for immunoblotting analysis using specific antibodies for the indicated proteins. Histograms show quantifications of the immunoblots performed by densitometry relative to GAPDH and presented as fold change relative to the control. Error bars represent mean ± SEM (n = 3). *P ≤ 0.05, **P ≤ 0.01, ns: not significant.

In order to assess the cytotoxic potency of PAC on anaplastic thyroid cancer cells, CAL-62 cells were treated either with DMSO (≤ 1%) as a control or with different concentrations of PAC (1, 3, 6 and 10 μM) for 72 h. The proportion of cell death (apoptosis + necrosis) were assessed by Annexin V/PI-flow cytometry, and the most effective PAC concentrations were determined. Figure 1A shows a dose-dependent increase in the proportion of apoptosi, reaching 53% and 61% upon treatment with 6 μM and 10 μM, respectively (Fig. 1A).

To investigate the cytotoxic effect of PAC over time on thyroid cancer cells, CAL-62 cells were treated with PAC (10 μM) at variable time periods 0, 24, 48 and 72 h, and the proportion of apoptotic cells was determined by the Annexin V/PI-Flow cytometry assay. Figure 1B shows the proportion and nature of cell death in CAL-62 treated with PAC (10 μM) at different time intervals. PAC induced apoptosis in a time-dependent manner, with the maximum proportion of apoptosis (60%) was reached after 72 h of treatment. These results indicate that PAC induces apoptosis in the highly resistant anaplastic CAL-62 cells in a time- and dose-dependent manner.

### PAC induces apoptosis in thyroid cancer cells through the mitochondrial pathway

To elucidate the molecular mechanism underlying PAC-dependent induction of apoptosis in thyroid cancer cells, CAL-62 cells were either sham-treated (DMSO) or challenged with PAC (10 μM) for 24, 48 and 72 h, and then cell lysates were prepared and protein levels were assessed by immunoblotting using specific antibodies, while GAPDH was used as internal control. Figure 1C shows that after 72 h of treatment, PAC increased the level of cleaved caspase 3 (12 fold) after 24 h of treatment and cleaved PARP-1 (fivefold) after 48 h of treatment. This confirms PAC-dependent induction of apoptosis in CAL-62 thyroid cancer cells. Next, we have shown PAC-dependent increase in the level of cleaved caspase 9 (15 fold) after 24 h of treatment, which indicates the induction of apoptosis through the mitochondrial pathway (Fig. 1C).

The results were confirmed by evaluating the levels of the pro- and anti-apoptotic proteins (Bax and Bcl-2, respectively). Figure 1D shows PAC-dependent upregulation of Bax (tenfold) after 48 h of treatment. However, the level of Bcl-2 was significantly reduced at 48 h (Fig. 1D). Consequently, PAC-treatment led to a significant increase in the Bax/Bcl-2 ratio reaching a level 20 fold higher after 48 h of treatment (Fig. 1D). These results indicate that PAC induces apoptosis mainly through the mitochondrial pathway.

### PAC inhibits the proliferation and migration abilities of thyroid cancer cells

**Figure 2:**
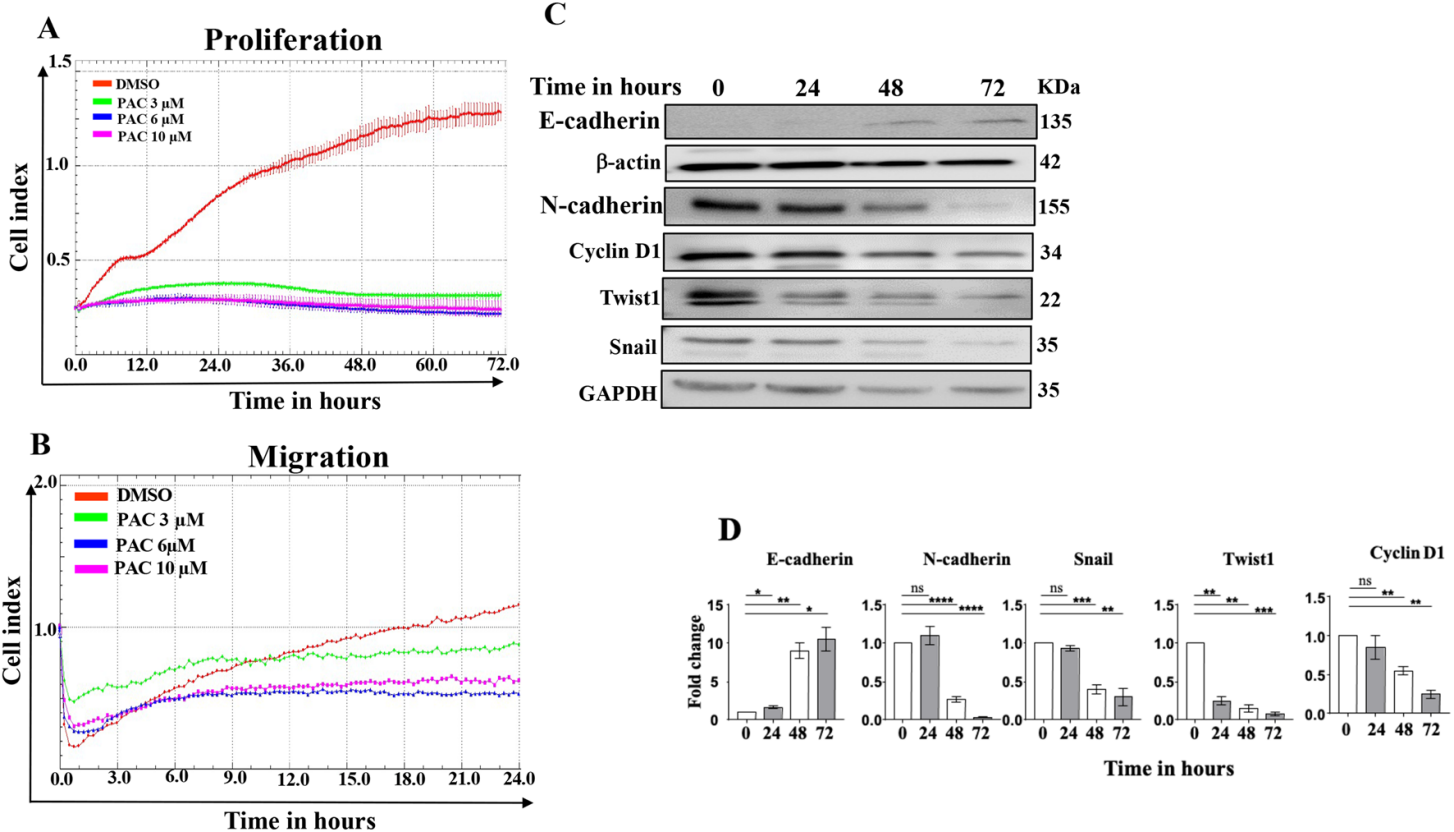
PAC suppresses the epithelial-to-mesenchymal transition processe in thyroid cancer cells. (**A**, **B**) CAL-62 cells were either sham-treated (DMSO ≤ 1%) or challenged with PAC (3, 6 or 10 µM) for the indicated periods of time. Exponentially growing cells (10^4^) were seeded independently in the E-plate (proliferation) or CIM plate (migration), and then the assessments were performed using the RTCA-DP xCELLigence System. Data are representative of different experiments performed in triplicate. (**C**) CAL-62 cells were treated with PAC (10 µM) for the indicated periods of time, and then cell lysates were prepared and were used for immunobloting analysis utilizing specific antibodies against the indicated proteins. (**D**) Histograms show quantifications of the immunoblots performed by densitometry relative to GAPDH and β-actin, and presented as fold change relative to the control. Error bars represent mean ± SEM (n = 3). *P ≤ 0.05, **P ≤ 0.01, ***P ≤ 0.001, ****P ≤ 0.0001, ns: not significant.

To assess the anti-proliferative effect of PAC on thyroid cancer cells, CAL-62 cells were treated with PAC (3, 6 and 10 μM) or DMSO used as control. Cell proliferation was measured over 72 h using the xCELLigence real-time cell analyzer (RTCA). Figure 2A shows that PAC at 3 μM and higher concentrations abolished cell proliferation ability of CAL-62 cells as compared to controls (DMSO). PAC-treated cells were fivefold less replicative than DMSO-treated cells (Fig. 2A). This anti-proliferative effect was confirmed at the molecular level by showing that PAC downregulated the cell cycle regulatory protein cyclin D1 (Fig. 2C). This indicates that PAC is a strong inhibitor of the proliferation of anaplastic thyroid cancer cells in vitro.

Next, CAL-62 cells were treated as described above: PAC (3, 6, 10 µM) or DMSO for 24 h, and then were seeded in a CIM-plate for another 24 h. Figure 2B shows that cells treated with DMSO continued migrating over time, while PAC treatment at 3 µM inhibited the migration ability of CAL-62 cells (1.5 fold), and PAC inhibitory effect was more pronounced at 6 and 10 µM reaching twofold and 2.4 fold inhibition over 24 h of treatment. This indicates that PAC suppresses the migration ability of thyroid cancer cells.

### PAC suppresses the epithelial-to-mesenchymal transition of thyroid cancer cells in vitro

Next, we tested the possible role of PAC in inhibiting the pro-metastatic epithelial-to-mesenchymal transition (EMT) process. To this end, CAL-62 cells were either sham-treated or challenged with PAC (10 μM) for 24, 48 and 72 h, and then cell lysates were used for immunoblotting analysis. Figure 2C,D shows that PAC upregulated the epithelial marker E-cadherin, while it reduced the levels of the mesenchymal markers (N-cadherin, Snail and Twist1). These results indicate that PAC suppresses the EMT process in thyroid cancer cells.

### PAC suppresses the mTOR pathway

**Figure 3:**
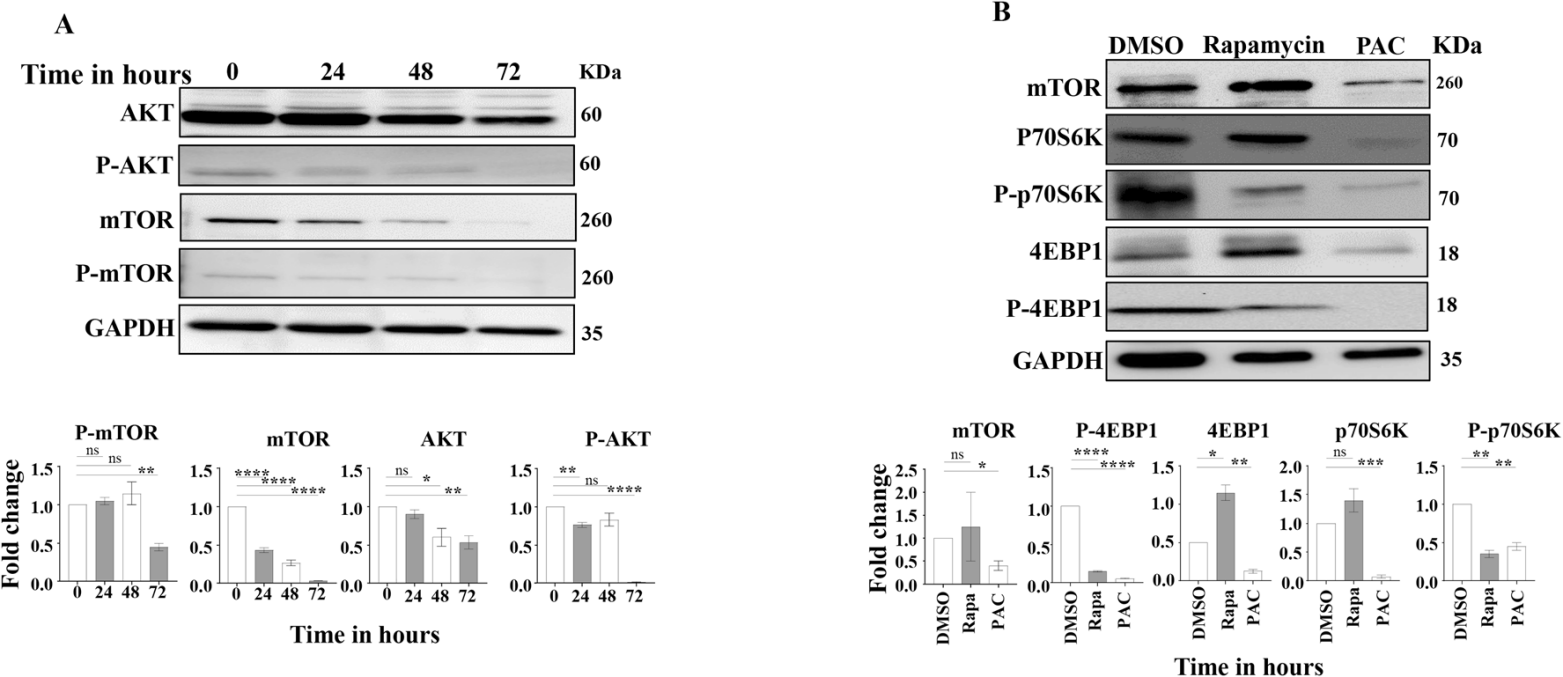
PAC suppresses the AKT/mTOR pathway in thyroid cancer cells. (**A**) CAL-62 cells were treated with PAC (10 µM) for the indicated periods of time, and then cell lysates were prepared and were used for immunebloting analysis utilizing specific antibodies against the indicated proteins. (**B**) CAL-62 cells were treated with PAC (10 µM) or rapamycin (50 nM) for 48 h, and then cell lysates were prepared and were used for immunobloting analysis utilizing specific antibodies against the indicated proteins. Histograms show quantifications of the immunoblots performed by densitometry relative to GAPDH, and presented as fold change relative to the control. The levels of phosphorylated proteins were normalized against the total amount of their relative non-phosphorylated forms. Error bars represent mean ± SEM (n = 2). *P ≤ 0.05, **P ≤ 0.01, ***P ≤ 0.001, ****P ≤ 0.0001, ns: not significant.

To investigate the potential role of PAC in inhibiting the AKT/mTOR pathway in thyroid cancer cells, CAL-62 cells were either sham-treated or exposed to PAC (10 μM), and then were harvested at different periods of time (24, 48 and 72 h). Cell lysates were prepared and were used for immunoblotting analysis. Figure 3A shows that PAC reduced the level of AKT with a higher effect observed on the phosphorylated form of the protein (pAKT) after 72 h of treatment. However, the effect on mTOR was clear only after 24 h of treatment (twofold decrease as compared to controls), and then the effect increased in a dose-dependent manner (Fig. 3A). Consequently, the level of the phosphorylated form of the protein was also reduced in a proportional menner, and then was halved after 72 h of treatment (Fig. 3A). These results indicate that PAC downregulates mTOR in thyroid cancer cells.

Next, we sought to study the inhibition efficacy of PAC as compared to rapamycin, a well known inhibitor of mTOR. To this end, CAL-62 cells were either sham-treated (DMSO) or exposed to PAC (10 μM) or rapamycin (50 μM) for 48 h. The immunoblotting analysis shows that while rapamycin had only slight effect on the level of mTOR, PAC downregulated mTOR more than twofold relative to the control (Fig. 3B). Furthermore, PAC had a stronger inhibitory effect than rapamycin on the expression of the mTOR downstream effectors P70S6K and 4EBP1 and their phosphorylated forms (Fig. 3B). This shows that PAC inhibits the mTOR pathway in anaplastic thyroid cancer cells.

### PAC suppresses the growth of thyroid tumor xenografts

**Figure 4:**
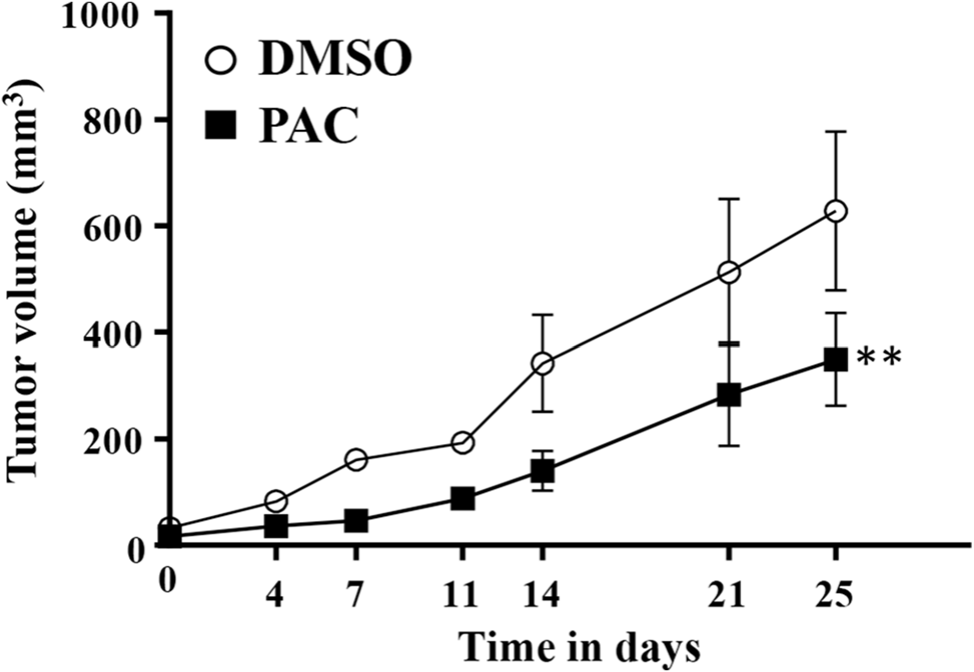
PAC inhibits the growth of human thyroid tumor xenografts. Thyroid cancer xenografts were created by subcutaneous injection of CAL-62 cells (5.10^6^) into nude mice (n = 6). Subsequent to the growth of the tumors, animals were randomized into two sub-groups. One group was PAC-treated intraperitonially at a dose of 100 µg/kg daily. The control group was treated with DMSO. The volumes of the tumors were measured at the indicated periods of time. The error bars represent means ± SEM, **P = 0.0085.

To investigate the potential role of PAC as therapeutic agent for thyroid cancer in vivo, thyroid cancer xenografts were created in nude mice by subcutaneous injection of CAL-62 cells (5 × 10^6^). When tumors reached a reasonable volume, the animals were randomized into two treatment sub-groups to receive intraperitoneal (i.p.) injections of PAC (100 µg/kg/day) or the same volume of dimethyl sulfoxide (DMSO) for 25 days. During this period tumor volume was measured regularly. Figure 4 shows time-dependent increase in the volume of the tumors in controls as well as PAC-treated animals. However, a clear and significant tumor growth inhibition was observed for animals treated with PAC as compared to those treated with DMSO (*P* = 0.0085) (Fig. 4). This shows PAC-dependent inhibition of tumor growth in vivo.

## Discussion

In the present study, we have shown the efficacy of the curcumin analogue PAC against anaplastic thyroid carcinoma cells both in vitro and in vivo. Indeed, PAC is cytotoxic and can also suppress the pro-metastatic EMT and stemness processes in thyroid cancer cells. These PAC anti-cancer properties were previously shown in different types of cancer cells^8,10–12^.

Interestingly, PAC treatment increased the expression of the epithelial marker E-cadherin and decreased the expression of the mesenchymal markers (N-cadherin, Snail and Twist1) (Fig. 2). It has been previously shown that modulation in the expression of E-cadherin affects the EMT process and the metastatic capacity of thyroid cancer cells^14^. Here, we have shown PAC-dependent E-cadherin upregulation and decrease in the migratory ability of ATC cells, which could result from increased cell–cell adhesion property^15,16^. PAC-related inhibition of the EMT process was further confirmed by showing PAC-dependent inhibition of the proliferative and the migratory abilities of ATC cells (Fig. 2A).

Furthermore, we have found that PAC reduced the level of the active form of the AKT protein kinase. In fact, Todaro et al.^17^ have shown that silencing of the AKT gene can block the metastatic capacity of the thyroid cancer stem cells^17^. Indeed, this pathway is responsible for maintaining the survival, proliferation, invasion, and migration of cancer cells^18^. PAC-induced inhibition of AKT prevented the phosphorylation/activation of its major target serine/threonine protein kinase mTOR (Fig. 3). This was confirmed by showing PAC-dependent inhibition of mTOR-mediated phosphorylation and activation of the downstream effector molecules p70S6K and 4E-BP1^18^. Cyclin D1 is another downstream effector molecule of the PI3K/AKT/mTOR signaling pathway. Cyclin D1 was down-regulated in response to PAC, which parallels previous findings^8,10–12^.

Interestingly, the AKT/mTOR pathway inhibitory effect of PAC was better than rapamycin (Fig. 3), a well-known mTOR inhibitor^19,20^. Therefore, PAC could be of great therapeutic value for ATC through the inhibition of one of the major caner pathways AKT/mTOR and the consequent inhibition of the pro-metastatic process EMT.

## Supplementary Information

Supplementary Figures.

## Data Availability

The data generated, used and analyzed in the current study are available from the corresponding author in response to reasonable request.

## Abbreviations

ATC: Anaplastic thyroid carcinoma
ATCC: American type culture collection
DMSO: Dimethyl sulfoxide
DTC: Differentiated thyroid cancers
EMT: Epithelial-to-mesenchymal transition
GAPDH: Glyceraldehyde-3-phosphate dehydrogenase
MTC: Medullary thyroid carcinoma
PAC: (3,5-*Bis* (4-hydroxy-3-methoxybenzylidene)-*N*-methyl-4-piperidone)
PBS: Phosphate buffered saline
