# Supplementary figures and images for: The curcumin analogue PAC has potent anti-anaplastic thyroid cancer effects
Source: Sci Rep. 2023 Mar 14;13:4217. doi: 10.1038/s41598-023-30888-2 (PMC10015015; doi:10.1038/s41598-023-30888-2)

**Figure 1C**

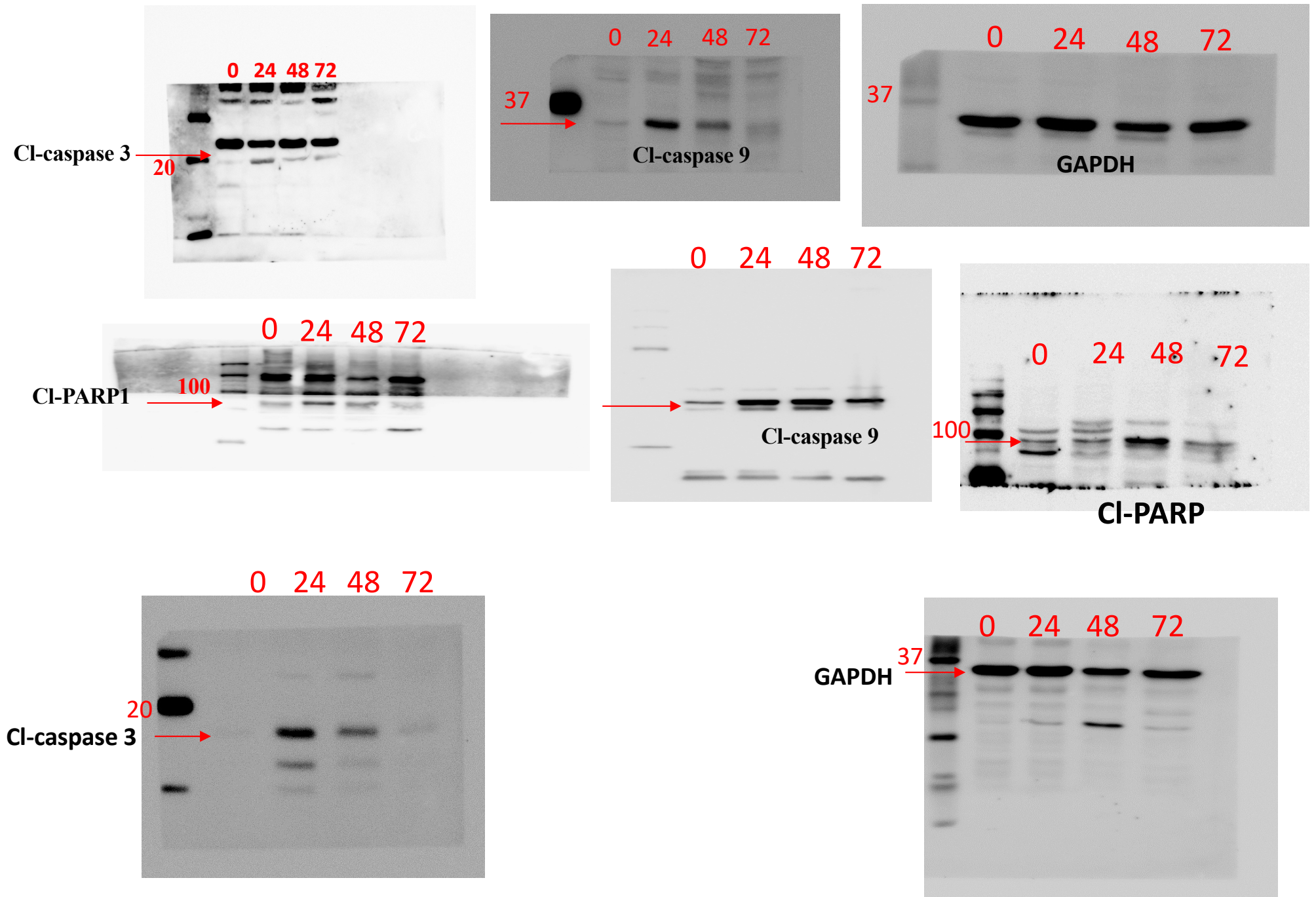

Figure 1

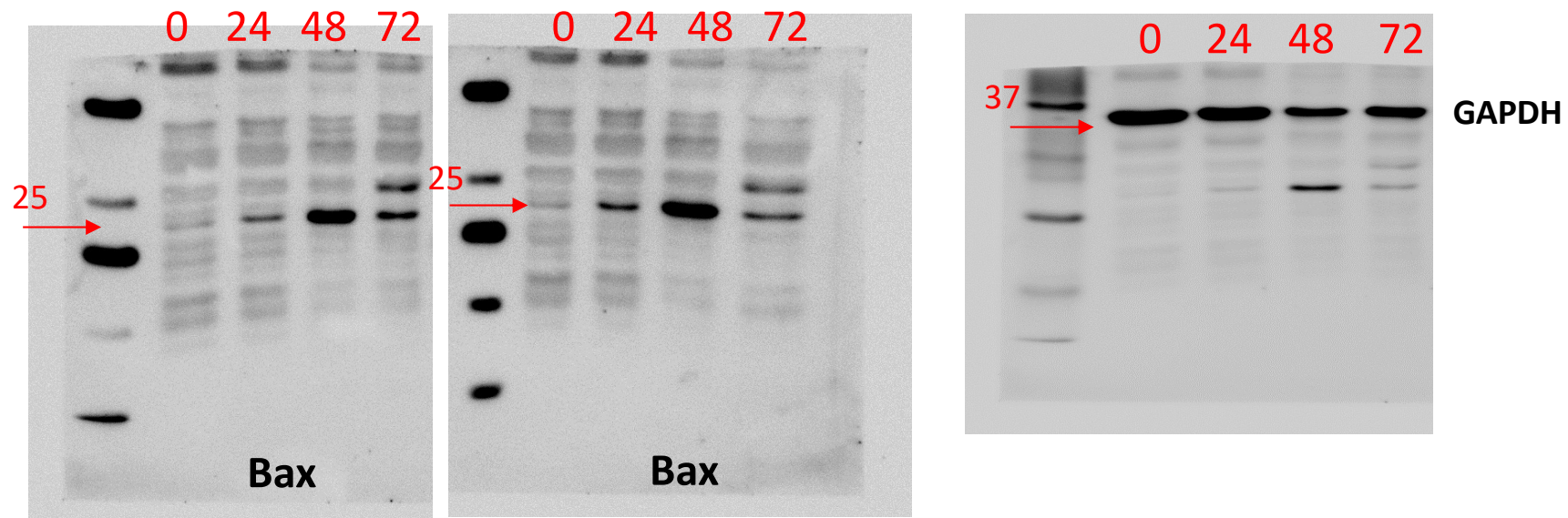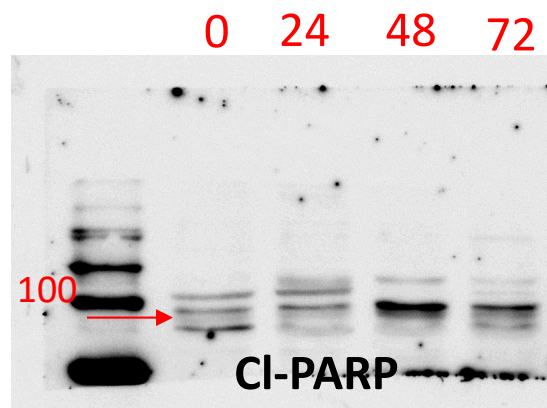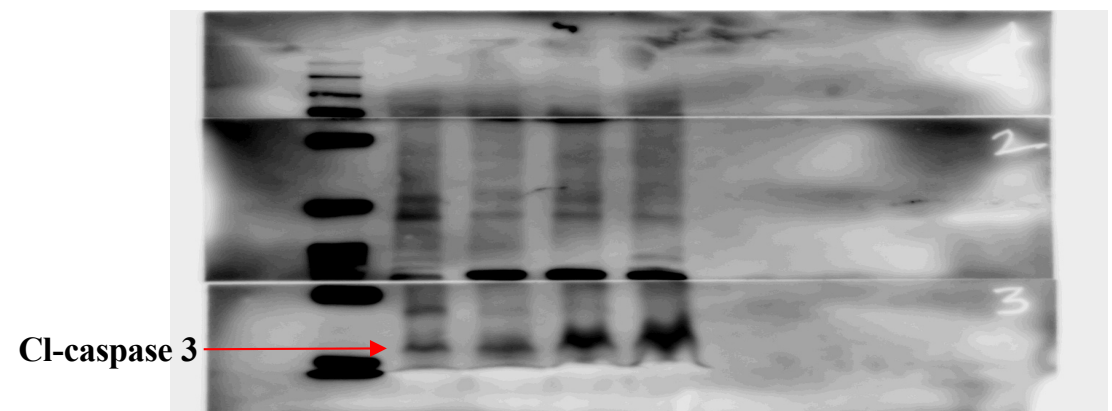

**Figure 1**

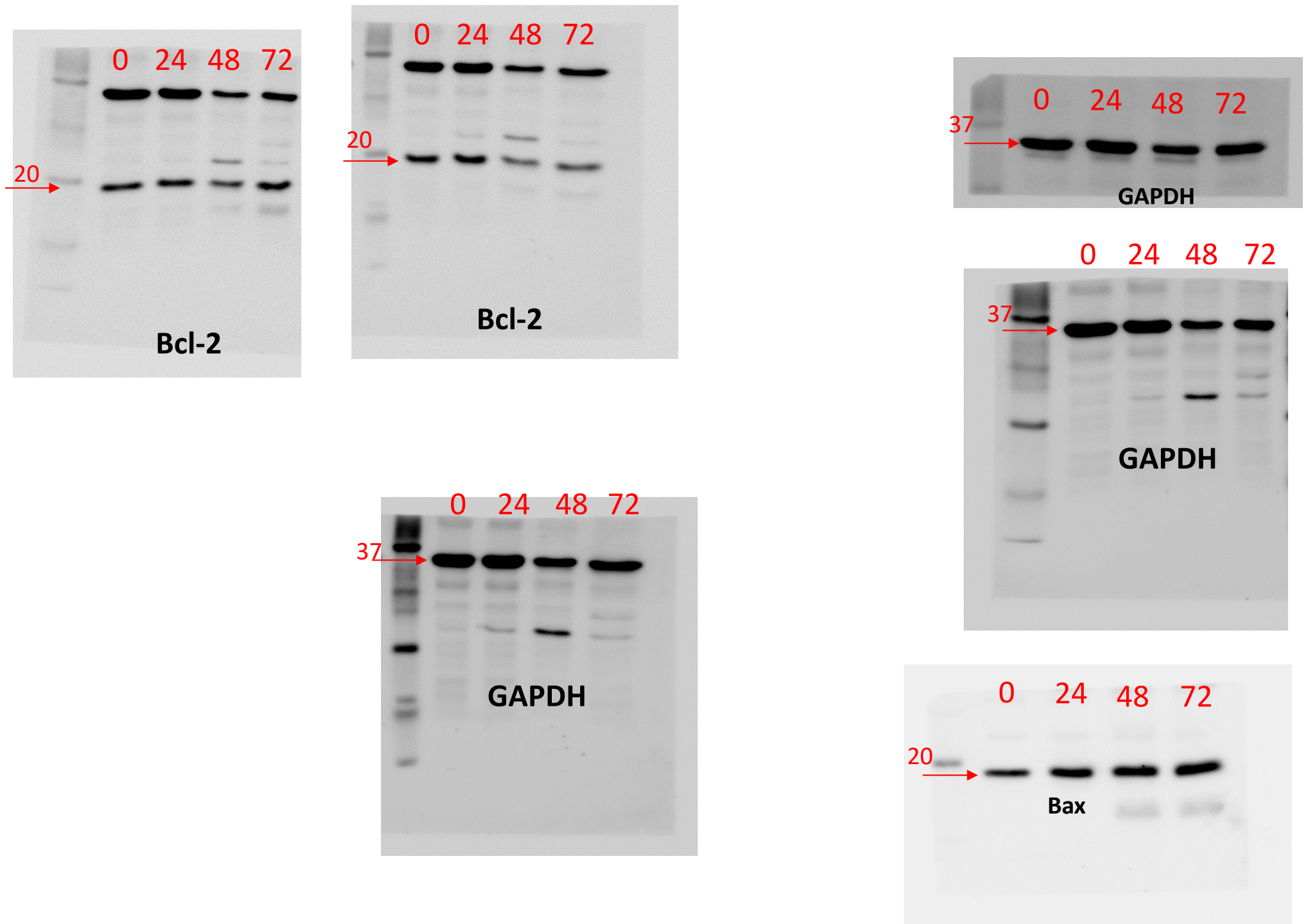

Figure 2C

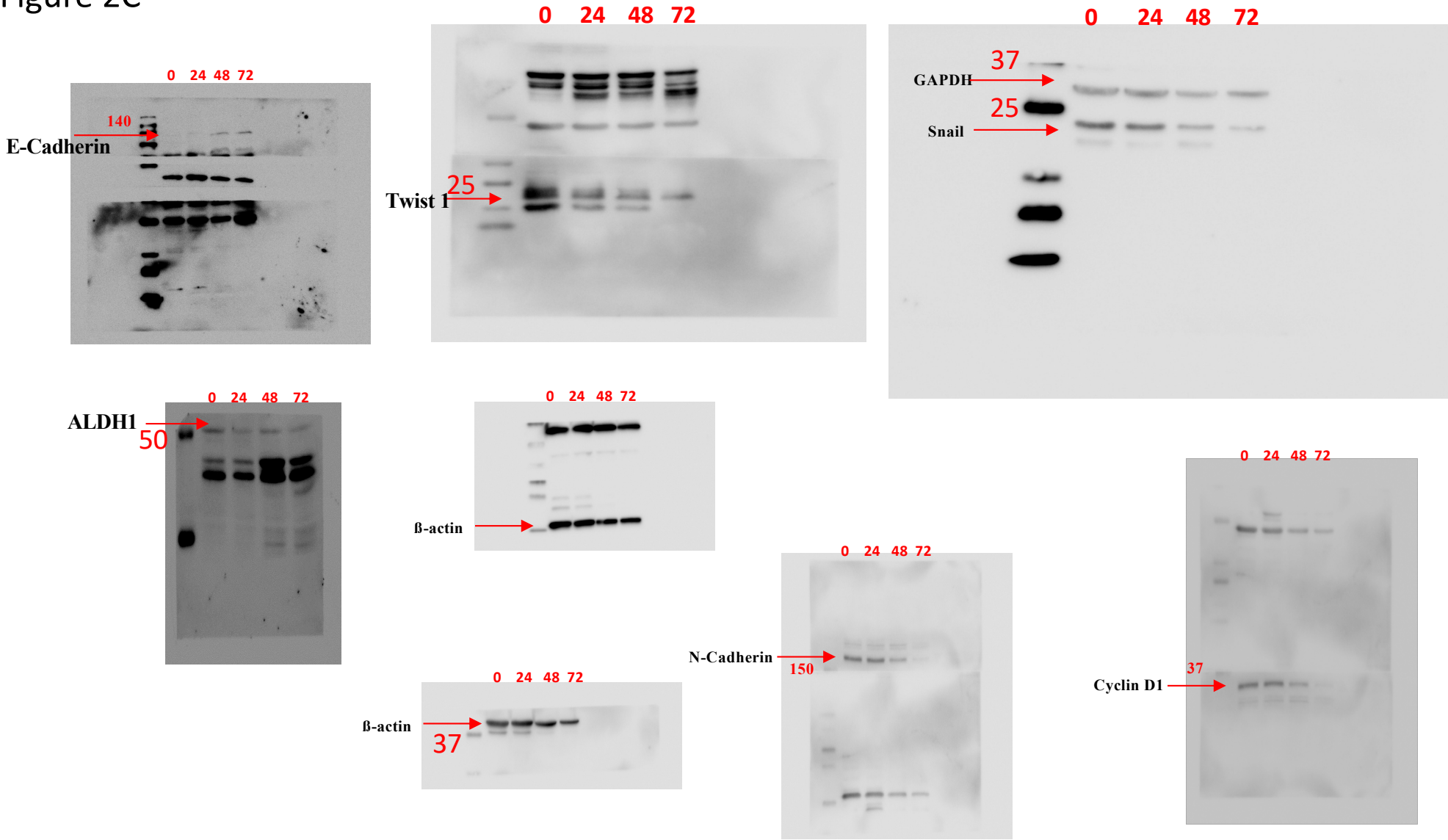

Figure 2C

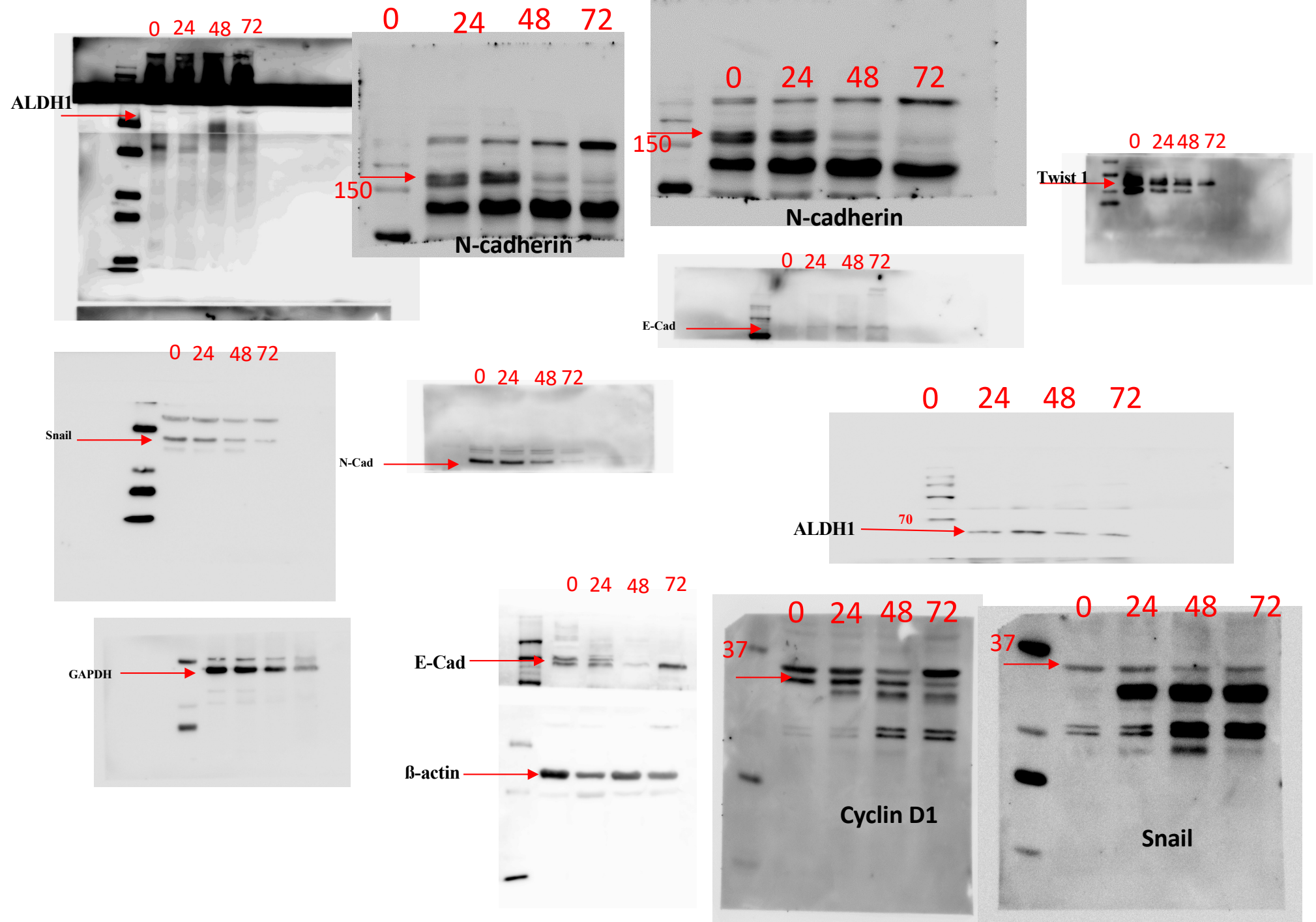

Figure 3A

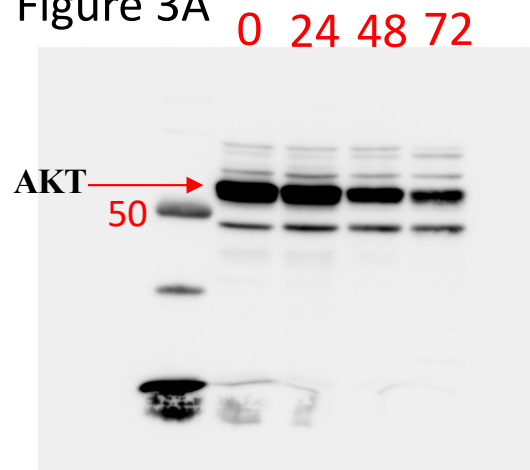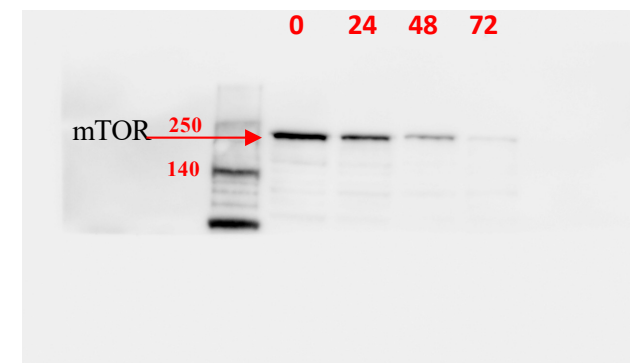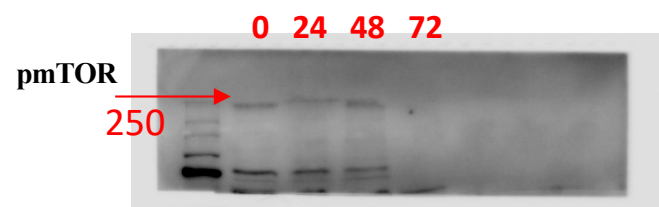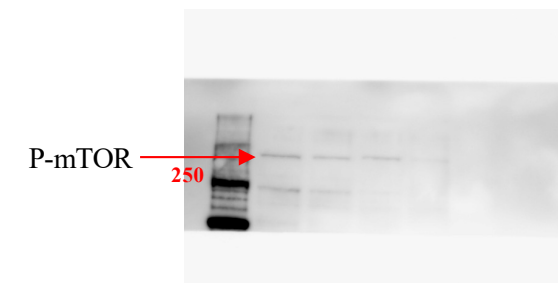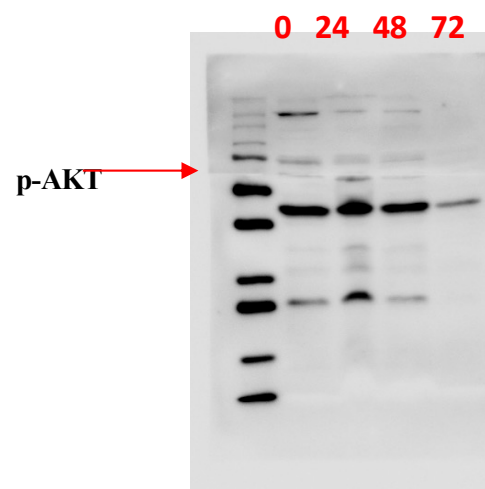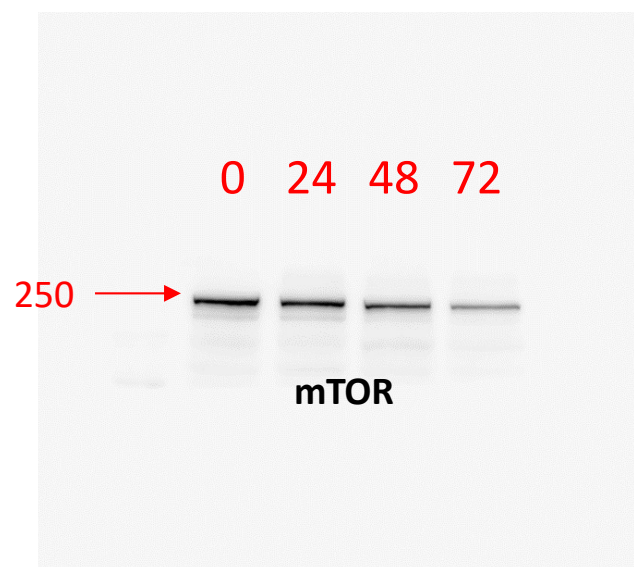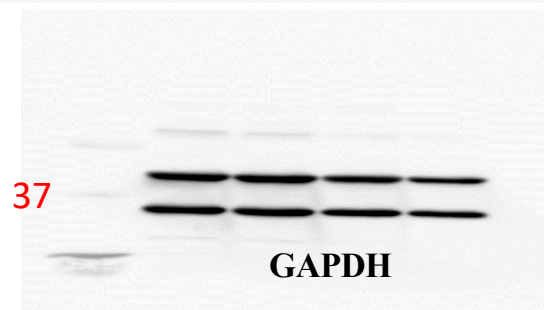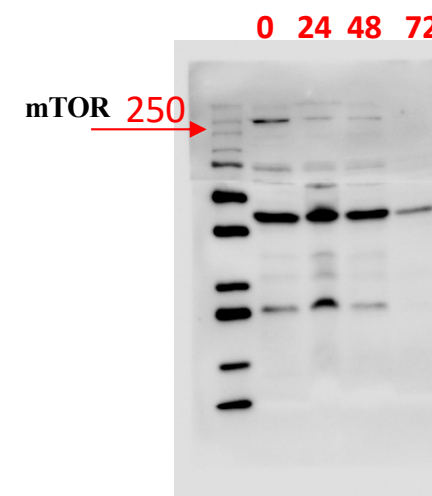

Figure 3A

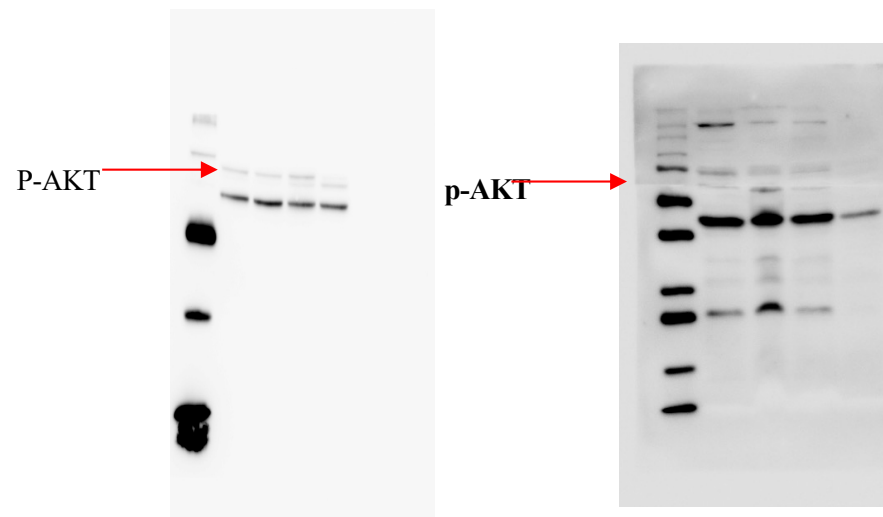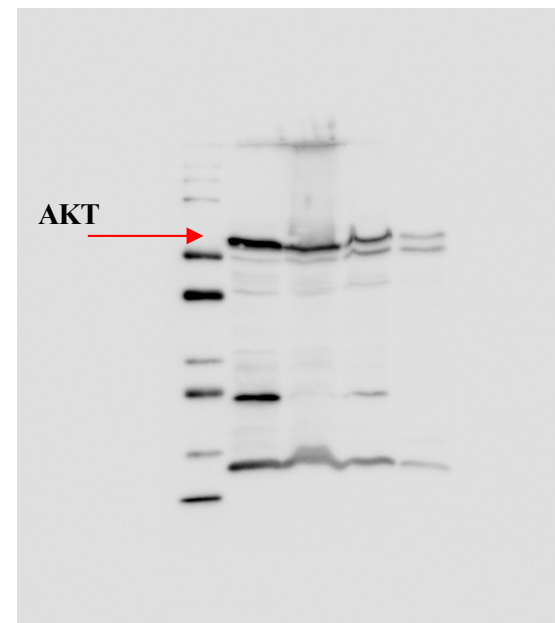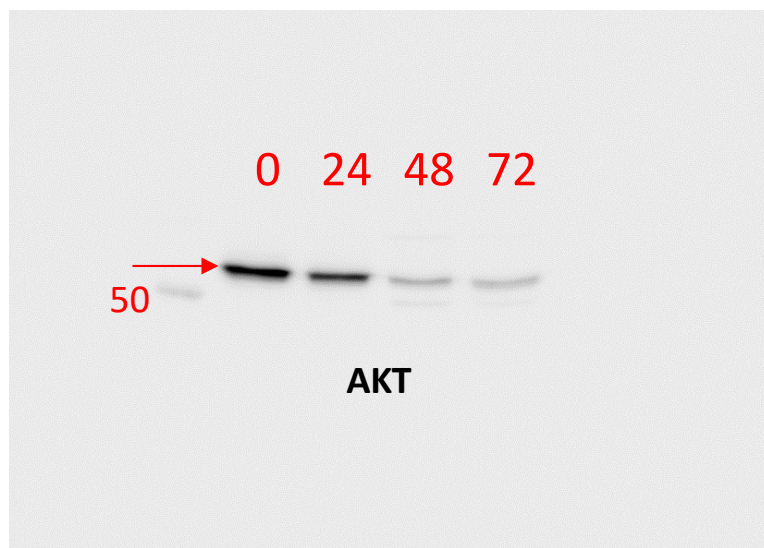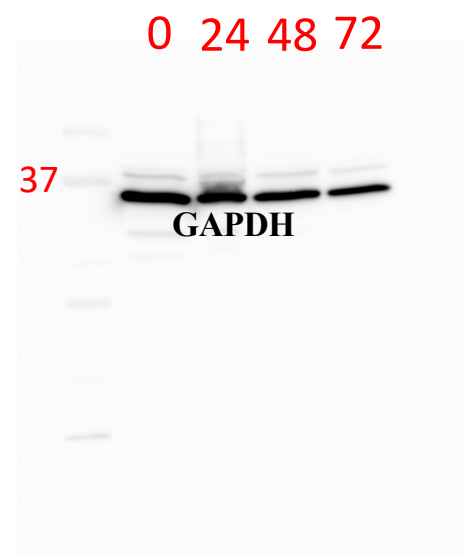

Figure 3B

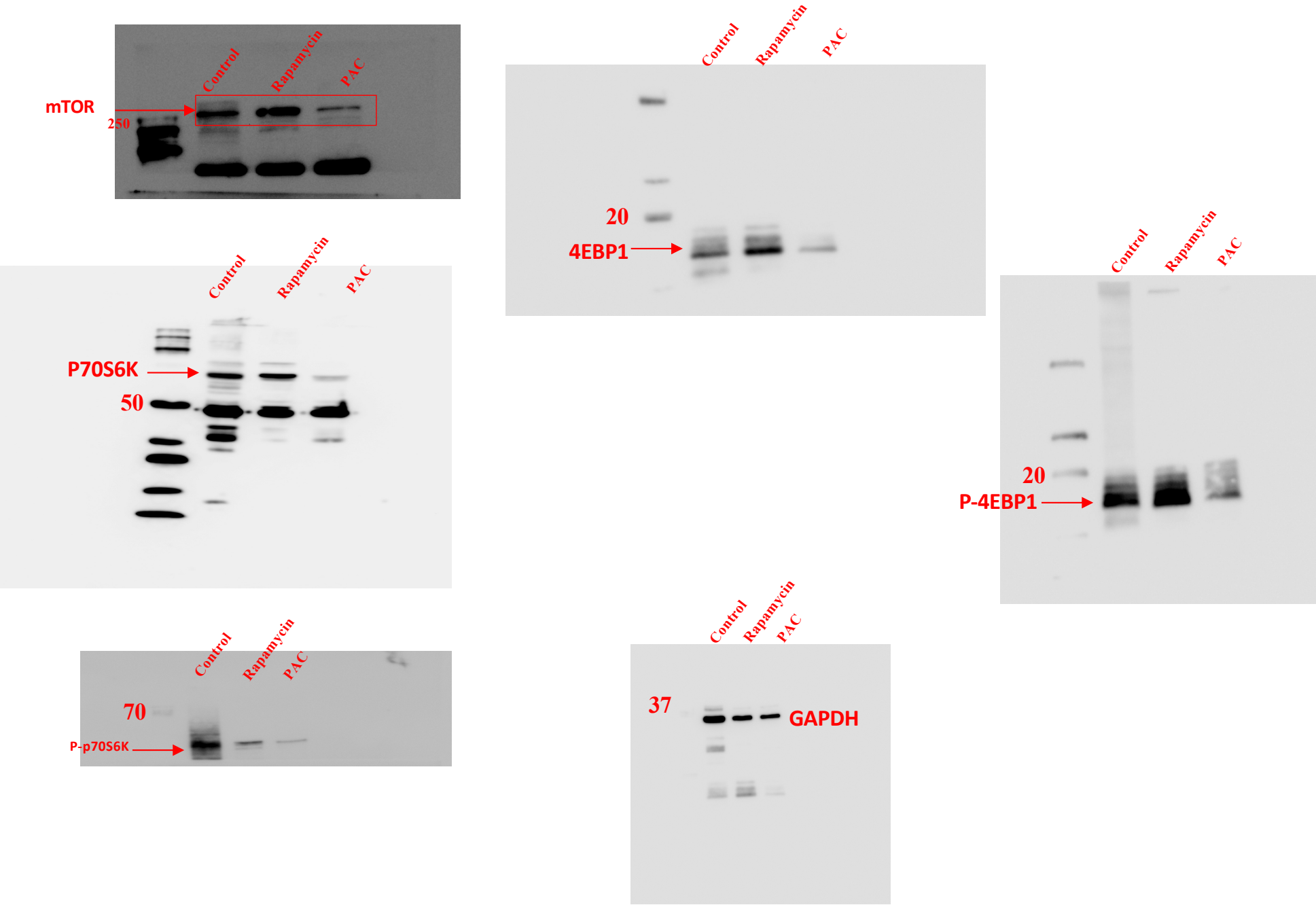

Figure 3B

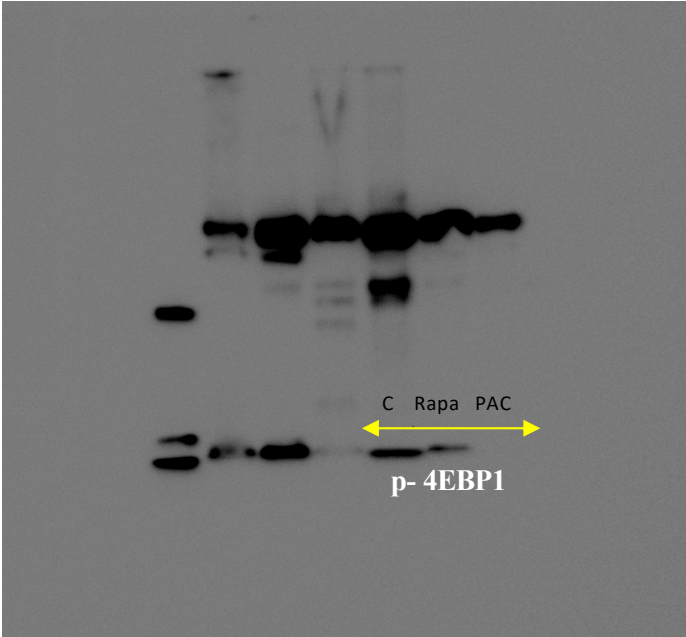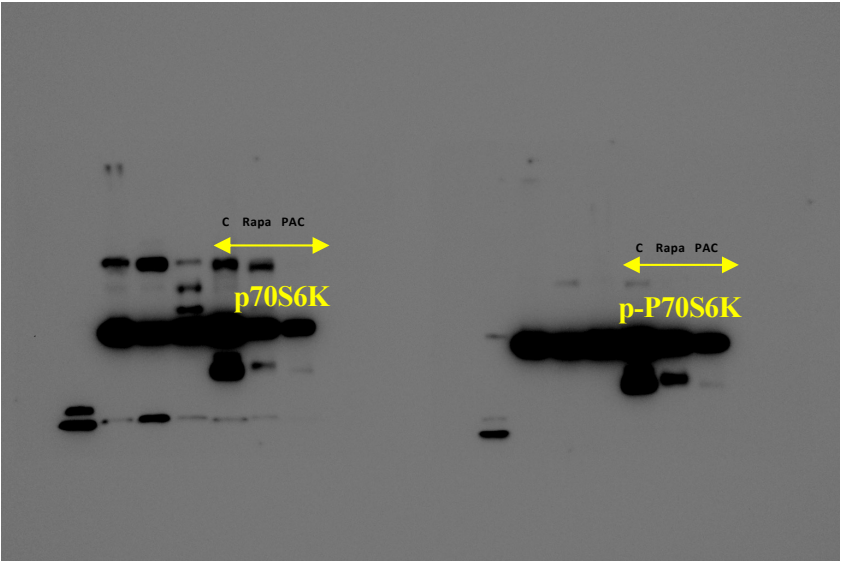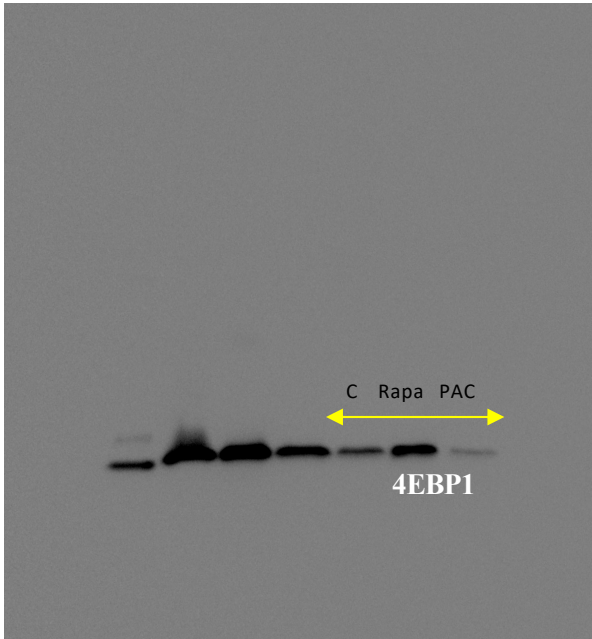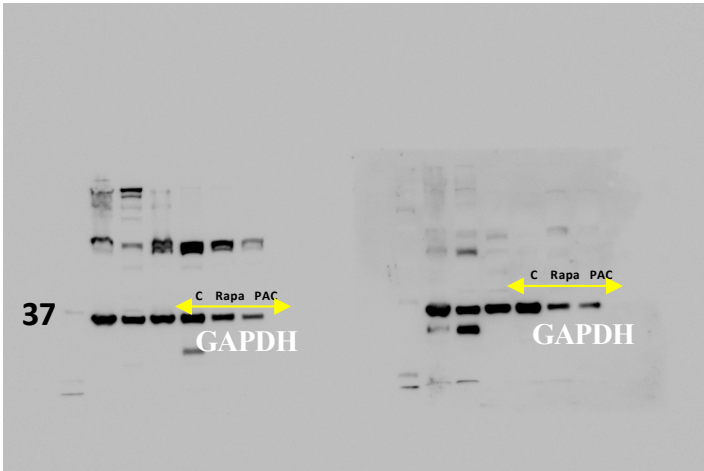

Supplement: Supplementary file 1 — Supplementary Figures. [file 41598_2023_30888_MOESM1_ESM.pdf]
